# Supplementary material for: Advanced siRNA Designs Further Improve In Vivo Performance of GalNAc-siRNA Conjugates
Source: Mol Ther. 2018 Jan 4;26(3):708–17. doi: 10.1016/j.ymthe.2017.12.021 (PMC5910670; doi:10.1016/j.ymthe.2017.12.021)

YMTHE, Volume *26*

Supplemental Information

Advanced siRNA Designs Further Improve *In Vivo* Performance of GalNAc-siRNA Conjugates

Donald J. Foster, Christopher R. Brown, Sarfraz Shaikh, Casey Trapp, Mark K. Schlegel, Kun Qian, Alfica Sehgal, Kallanthottathil G. Rajeev, Vasant Jadhav, Muthiah Manoharan, Satya Kuchimanchi, Martin A. Maier, and Stuart Milstein

Supplementary Information

Advanced siRNA designs further improve *in vivo* performance of GalNAc-siRNA conjugates

Donald J Foster, Christopher R Brown, Sarfraz Shaikh, Casey Trapp, Mark K. Schlegel, Kun Qian, Alfica Sehgal, Kallanthottathil G. Rajeev, Vasant Jadhav, Muthiah Manoharan, Satya Kuchimanchi, Martin A. Maier, and Stuart Milstein[*]

Content

Supplementary Table 1. Sequence information of siRNAs used in this work

Supplementary Figure 1. Alternate analysis of optimization of sense and antisense strand designs

Supplementary Table 1

Target	Sequence	Strand	Sequence (5' – 3')
Mouse *Ttr* (NM_013697.5)	1	S	AACAGUGUUCUUGCUCUAUAA
		AS	UUAUAGAGCAAGAACACUGUUUU
Mouse *Ttr* (NM_013697.5)	2	S	AAACAGUGUUCUUGCUCUAUA
		AS	UAUAGAGCAAGAACACUGUUUUG
Mouse *Ttr* (NM_013697.5)	3	S	CUUGCUCUAUAAACCGUGUUA
		AS	UAACACGGUUUAUAGAGCAAGAA
Mouse *Ttr* (NM_013697.5)	4	S	UCCUCUGAUGGUCAAAGUCCU
		AS	AGGACUUUGACCAUCAGAGGACA
Mouse *Cfb* (NM_008198.2)	5	S	GAUUGAGAAGGUGGCGAGUUA
		AS	UAACUCGCCACCUUCUCAAUCAA
Mouse *Cfb* (NM_008198.2)	6	S	CACAGAGAAGCUCAACCAAAU
		AS	AUUUGGUUGAGCUUCUCUGUGAC
Mouse *Cfb* (NM_008198.2)	7	S	UUGUGAGAGAGAUGCUACAAA
		AS	UUUGUAGCAUCUCUCUCACAACU
Mouse *Agt* (NM_007428.3)	8	S	GCGCUGAAGGAUACACAGAAA
		AS	UUUCUGUGUAUCCUUCAGCGCCA
Mouse *Agt* (NM_007428.3)	9	S	ACUGCGCUGACCGAGAAUAAA
		AS	UUUAUUCUCGGUCAGCGCAGUCU
Mouse *Agt* (NM_007428.3)	10	S	CCGAGUGGGAGAGGUUCUCAA
		AS	UUGAGAACCUCUCCCACUCGGGG

Supplementary Figure 1. Alternate analysis of optimization of sense and antisense strand designs. Impact relative to DV5 (A, B) or DV12 (C) is defined as the model-adjusted mean difference in activity of design variant (DV) compared to reference, and is presented in natural log.

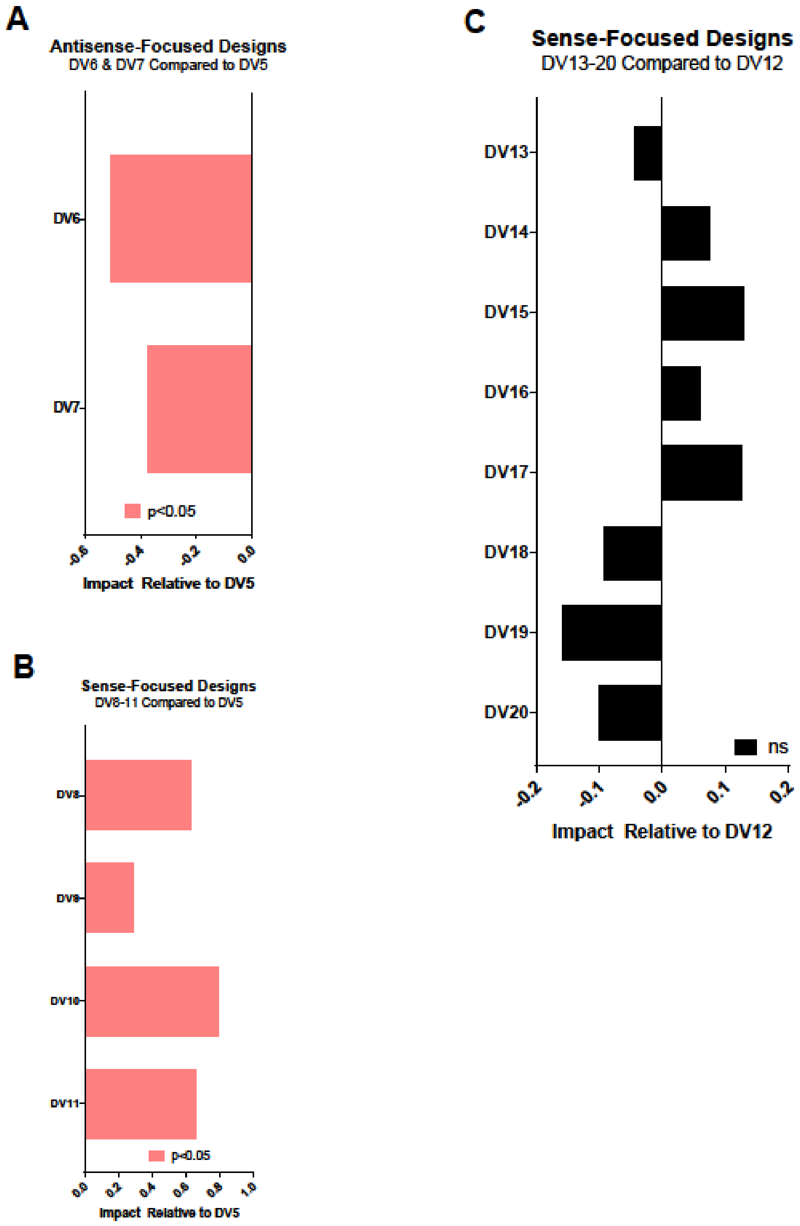

Supplement: Document S1. Figure S1 and Table S1 [file mmc1.pdf]
