# Supplementary material for: Advanced siRNA Designs Further Improve In Vivo Performance of GalNAc-siRNA Conjugates
Source: Mol Ther. 2018 Jan 4;26(3):708–17. doi: 10.1016/j.ymthe.2017.12.021 (PMC5910670; doi:10.1016/j.ymthe.2017.12.021)

Molecular Therapy
Original Article

Advanced siRNA Designs Further Improve *In Vivo* Performance of GalNAc-siRNA Conjugates

Donald J. Foster,[1] Christopher R. Brown,[1] Sarfraz Shaikh,[1] Casey Trapp,[1] Mark K. Schlegel,[1] Kun Qian,[1] Alfica Sehgal,[1] Kallanthottathil G. Rajeev,[1] Vasant Jadhav,[1] Muthiah Manoharan,[1] Satya Kuchimanchi,[1] Martin A. Maier,[1] and Stuart Milstein[1]

[1]Alnylam Pharmaceuticals, Cambridge, MA 02142, USA

Significant progress has been made in the advancement of RNAi therapeutics by combining a synthetic triantennary *N*-acetylgalactosamine ligand targeting the asialoglycoprotein receptor with chemically modified small interfering RNA (siRNA) designs, including the recently described Enhanced Stabilization Chemistry. This strategy has demonstrated robust RNAi-mediated gene silencing in liver after subcutaneous administration across species, including human. Here we demonstrate that substantial efficacy improvements can be achieved through further refinement of siRNA chemistry, optimizing the positioning of 2′-deoxy-2′-fluoro and 2′-*O*-methyl ribosugar modifications across both strands of the double-stranded siRNA duplex to enhance stability without compromising intrinsic RNAi activity. To achieve this, we employed an iterative screening approach across multiple siRNAs to arrive at advanced designs with low 2′-deoxy-2′-fluoro content that yield significantly improved potency and duration in preclinical species, including non-human primate. Liver exposure data indicate that the improvement in potency is predominantly due to increased metabolic stability of the siRNA conjugates.

INTRODUCTION

The endogenous RNAi pathway can be harnessed to inhibit the expression of genes through post-transcriptional gene silencing using synthetic small interfering RNAs (siRNAs).[1–3] The significant progress made in recent years has fueled the advancement of RNAi therapeutics as a promising new class of investigational medicines.[4–7] The conjugation of chemically modified, metabolically stable siRNAs to a synthetic triantennary *N*-acetylgalactosamine (GalNAc) ligand, represents a promising approach for safe and effective targeted delivery of RNAi therapeutics to hepatocytes *in vivo*.[8] The triantennary GalNAc ligand is designed to bind with high affinity and specificity to the asialoglycoprotein receptor (ASGPR), a hepatocellular transmembrane glycoprotein that is highly expressed on hepatocytes, which binds and rapidly removes desialylated glycoproteins from circulation.[9–13]

Aiming to improve the efficacy and potency of GalNAc-siRNA conjugates, we previously described the incorporation of chemical modifications that provide enhanced stabilization against nuclease activity.[14] This design combines 2′-*O*-methyl (2′-OMe) and 2′-deoxy-2′-fluoro (2′-F) ribosugar modifications throughout both strands of the siRNA with terminal phosphorothioate (PS) linkages, which provide additional protection against 3′ and 5′ exonucleases. Enhanced Stabilization Chemistry (ESC) conjugates exhibit median effective dose (ED_{50}) levels of ≤ 1 mg/kg following a single subcutaneous (s.c.) dose. Chronic weekly s.c. dosing of a GalNAc-siRNA conjugate in rodents resulted in sustained, dose-dependent gene silencing for over 9 months without any adverse effects.[14]

Aided by a favorable efficacy profile, the ESC GalNAc-siRNA platform has rapidly progressed into preclinical and clinical development to target hepatic genes of therapeutic interest.[15,16] Recent studies have shown that the improved potency and duration observed for ESC conjugates compared to the Standard Template Chemistry (STC) design were mainly driven by improvements in metabolic stability against 5′ exonuclease attack.[17]

It is well known in the field that modifying the 2′ position of RNA can significantly enhance the nuclease stability of oligonucleotides and that sterically more demanding modifications, such as 2′-OMe, can have a greater stabilizing effect compared to less bulky modifications, such as 2′-F.[18,19] If not applied judiciously, however, the steric bulk introduced by such modifications can substantially reduce RNAi activity.[20–22] We reasoned that further improvements in metabolic stability, particularly against endonucleolytic activity, could be achieved through an increase of 2′-OMe ribonucleotide content with a concomitant decrease in 2′-F ribosugar moieties. However, for the changes to have a positive impact on activity, the novel designs must maintain their ability to interact with the RNAi-induced silencing complex (RISC) for maintaining intrinsic activity.[21,23,24]

While fully PS-modified antisense oligonucleotides containing 2′-F ribosugar moieties have been observed to increase double-strand DNA breaks and impair cellular proliferation, evaluation of 2′-F in

Received 3 August 2017; accepted 24 December 2017;
https://doi.org/10.1016/j.ymthe.2017.12.021.

Correspondence: Stuart Milstein, PhD, 300 Third Street, Cambridge, MA 02142, USA.
E-mail: smilstein@alnylam.com

This is an open access article under the CC BY license (http://creativecommons.org/licenses/by/4.0/).

the context of siRNA has revealed no such liability.[21,23–25] Specifically, the liabilities identified by Shen et al.[26] appear to be specific to single-stranded antisense oligonucleotides with high PS content.[23] Additionally, clinical trial data have shown that 2′-F-containing siRNA are generally well tolerated.[25,27] To guide the refinement of siRNA chemistry through optimal positioning of 2′-F and 2′-OMe ribosugar moieties in both sense and antisense strands, we applied statistical analyses to a dataset of siRNA *in vitro* activity across a number of sequences. *In silico* analysis was followed by directed structure activity relationship (SAR) studies applied to a panel of model sequences. Our results indicate that significant improvements in potency and duration can be achieved with the new designs and that those improvements were attributable to increased metabolic stability.

RESULTS

The objective of this work was to optimize the number and placement of 2′-F and 2′-OMe residues for maximal stability, in other words, trying to minimize the overall number of 2′-F residues while maintaining or improving intrinsic activity. As a starting point for these experiments, we utilized a previously reported siRNA design.[14,17] The optimization strategy was based on an iterative *in vitro* and *in vivo* screening process to assess novel designs.

Permutations to the parent design were evaluated based on predictions from an *in silico* model generated by multiple linear regression. Using an *in vitro* silencing dataset of 1,890 duplexes with varying 2′-F and 2′-OMe composition across five targets and 15 target sites, an initial model describing the impact of 2′-F relative to 2′-OMe at each position in the antisense and sense strands was generated (Figures 1A and 1B). By analyzing this panel of different sequences, we hoped to minimize sequence-specific effects and identify sequence-agnostic design elements. However, due to the complexity of the design space even for two 2′ modifications (with 2^{21} and 2^{23} possible permutations for sense and antisense, respectively), the results from this analysis were considered starting points for further optimization rather than general design rules.

We found that our model fit the data well, with an adjusted r-squared value of 0.66 and $p < 2.2e{-}16$. From this analysis of the initial dataset, we identified certain positions in each strand that appear to be significantly impacted (positively or negatively) by 2′-F relative to 2′-OMe. Relative to the 5′ end of the respective strand, antisense strand positions 2, 6, and 14 (AS2, AS6, and AS14) as well as sense strand position 11 (S11) were found to exhibit the highest apparent preference for 2′-F.

To test the validity of the positional model, design variants (DVs) containing 2′-OMe rather than 2′-F at each of these critical positions in both strands (DVs 1–4) were made utilizing four siRNAs targeting the mouse transthyretin (*Ttr*) gene (Table S1, sequences 1–4) and evaluated *in vitro* (Figures 1C and 1D). The impact of each DV relative to parent is expressed as the model-adjusted mean value based on results from all individual sequences. In other words, each value represents the average effect of a given design across all sequences evaluated, and only the chemical modification pattern is shown for each DV. As expected from the modeling of the initial dataset, replacement of 2′-F with 2′-OMe at those positions generally led to a loss of activity. Differences in magnitude of effect for these permutations, compared with expectations from the model, may be due to the heterogeneous nature of the modeled dataset relative to the number of possible permutations across the 21/23-mer design. For example, while the model showed the most significant impact of 2′-F at AS14, the experimental results across the four sequences tested indicated that AS2 carried the greatest improvement when 2′-F was incorporated. AS6 showed a trend toward being impactful, but it did not meet the threshold for statistical significance. Position S11, as predicted by the model, showed a similar impact of 2′-F as AS14.

With the goal of maintaining *in vitro* activity while reducing 2′-F content, and having confirmed individual positions that require 2′-F, we next applied the *in silico* model to optimize the antisense strand design (Figure 2A). In addition to the critical positions mentioned above, the model predicted a negative impact of 2′-F at AS8 but a positive effect at AS9, with little effect on AS positions 4, 10, 18, and 20. To assess the optimal modification pattern at AS8 and AS9, three DVs were generated and evaluated against the four mouse *Ttr* sequences *in vitro*. Based on the model, 2′-F was retained at AS2, AS6, and AS14 and replaced with 2′-OMe at positions AS4, AS10, AS18, and AS20 across all three designs. For DV 5, the chemical modification pattern of the antisense strand was strictly based on the model with 2′-OMe at AS8 and 2′-F at AS9, while the two variants DV 6 and DV 7 contained a pair of 2′-OMe or 2′-F modifications at AS8 and AS9, respectively. Data were analyzed by regression to compare across sequences to parent (Figure 2A) as well as relative to DV 5 (Figure S1A). Surprisingly, DV 6 and DV 7 were both found to be more active compared to DV 5, and they maintained the same level of activity as the parent while DV 5 did not. Based on these results, we concluded that inclusion of either a pair of 2′-F or 2′-OMe at AS8 and AS9 is advantageous, which ultimately allowed for the reduction of the 2′-F content in the AS strand from nine to six (DV 7) and four (DV 6), respectively.

Given discrepancies between modeling and experimental results, we hypothesized that the effect of 2′ modifications at certain positions may be sensitive to the context in which they are placed, i.e., the overall modification pattern of both strands of the siRNA. Thus, we next evaluated four model-derived sense strand variants in the context of the model-derived antisense strand design (from DV 5). Based on the statistical modeling, 2′-F was consistently replaced by 2′-OMe at sense strand positions S1, S3, S5, S17, S19, and S21, while interrogating some of the other sense strand positions (S6, S7, S9, S10, S12, S13, and S15) with DVs 8–11 (Figure 2B). Interestingly, all designs resulted in a significant loss of activity compared to the parent, including DV 11 with only 2′-F residues at positions S6 and S11, predicted by the statistical model as the minimal 2′-F requirement for the sense strand. In a separate analysis, we found that DVs 8–11 also lost activity relative to DV 5, which shares a common antisense strand used as the starting point for this set of designs (Figure S1B).

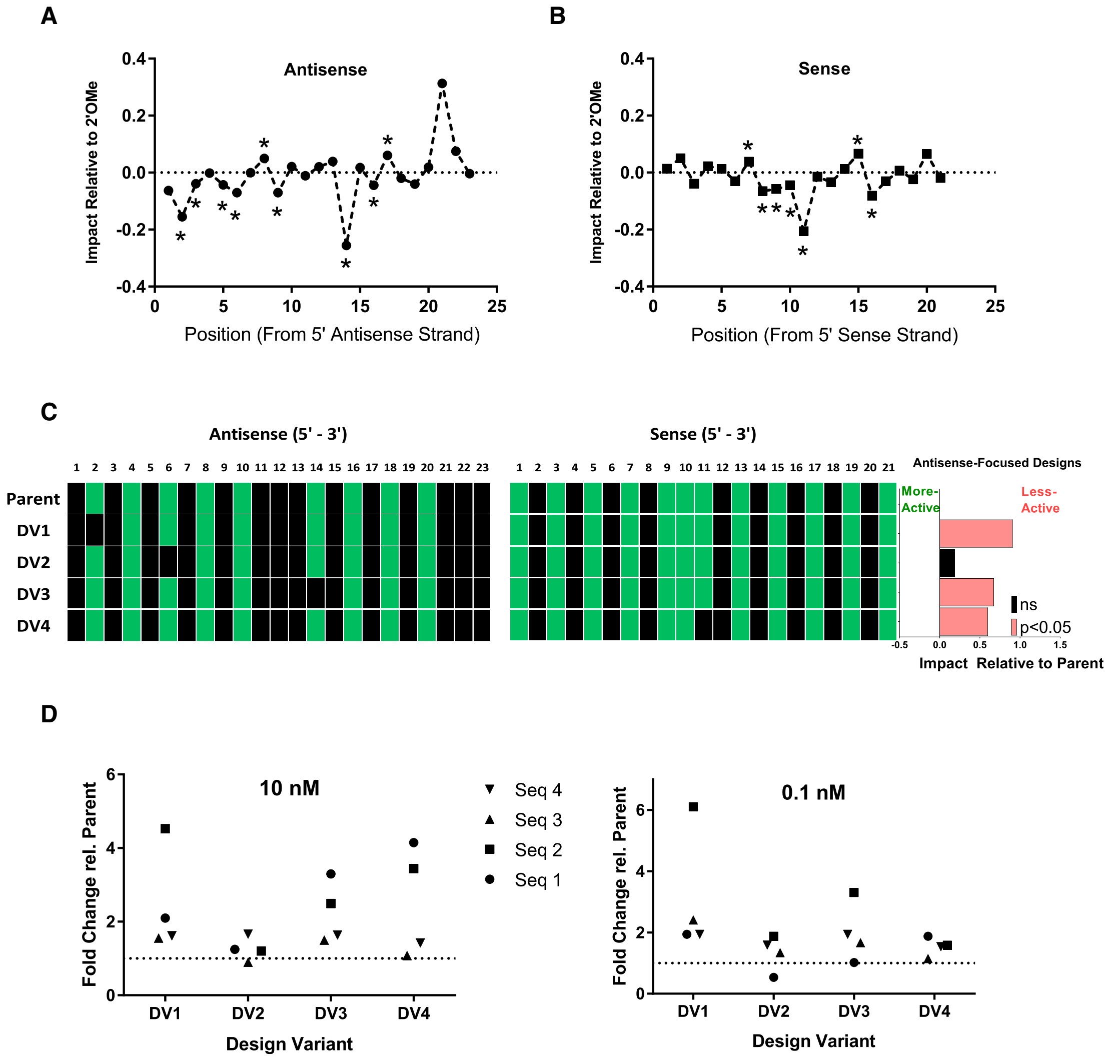

Figure 1. Relative Impact of 2′-F at Each Position in the Antisense and the Sense Strands Based on a Multiple Linear Regression Model

The y axis represents model-adjusted mean difference in target silencing, in natural log, of 2′-F from 2′-OMe-containing duplexes at a given position. (A and B) The x axis represents nucleotide position in the duplex, relative to the 5′ antisense (A) or 5′ sense strand (B). Negative numbers indicate activity improvement with inclusion of 2′-F relative to 2′-OMe at that position, positive numbers reflect decreased activity. Asterisks (*) indicate significant differences between 2′-F and 2′-OMe at the noted positions ($p < 0.05$). (C and D) Confirmation of effect *in vitro* across four siRNAs targeting the mouse transthyretin (*Ttr*) gene is depicted as fold-change relative to parent at either 10 nM (A) or 0.1 nM (B). 2′-F and 2′-OMe modifications are depicted as green and black squares, respectively.

To investigate to what extent the apparent loss in activity was due to a suboptimal antisense strand design, the most active of these initial sense strand designs (from DV 9) was paired with the antisense design from DV 7, resulting in a new DV (DV 12). As shown in Figure 2B, activity was restored almost to the level of the parent compound with substantially reduced 2′-F content. Starting from this new template DV 12, a series of sense strand designs was generated to systematically assess the impact of chemistry change at positions S1, S3, S5, S13, S15–17, and S19 in the sense strand relative to parent (DV 13–20). For example, sense position 1 in DV 13 was changed to 2′-F to match the parent design at that position. With the exception of DV 13, all other DVs showed activity similar to the parent. However, by reverting the chemistry at S16 from 2′-F to 2′-OMe, we were able to retain (relative to parent) and minimize the 2′-F content in the sense strand to four 2′-F ribosugar moieties (DV 18). In an analysis comparing DVs 13–20 to DV 12, all designs were similar to DV 12 with the exception of DV 19, which showed a trend ($p = 0.067$) toward improved activity (Figure S1C).

Next, we evaluated the two most active sense and antisense strand variants *in vitro* in a combinatorial fashion. To confirm the generalizability of the designs, the screening panel was expanded to a total of ten different sequences including the four previously used mouse *Ttr*

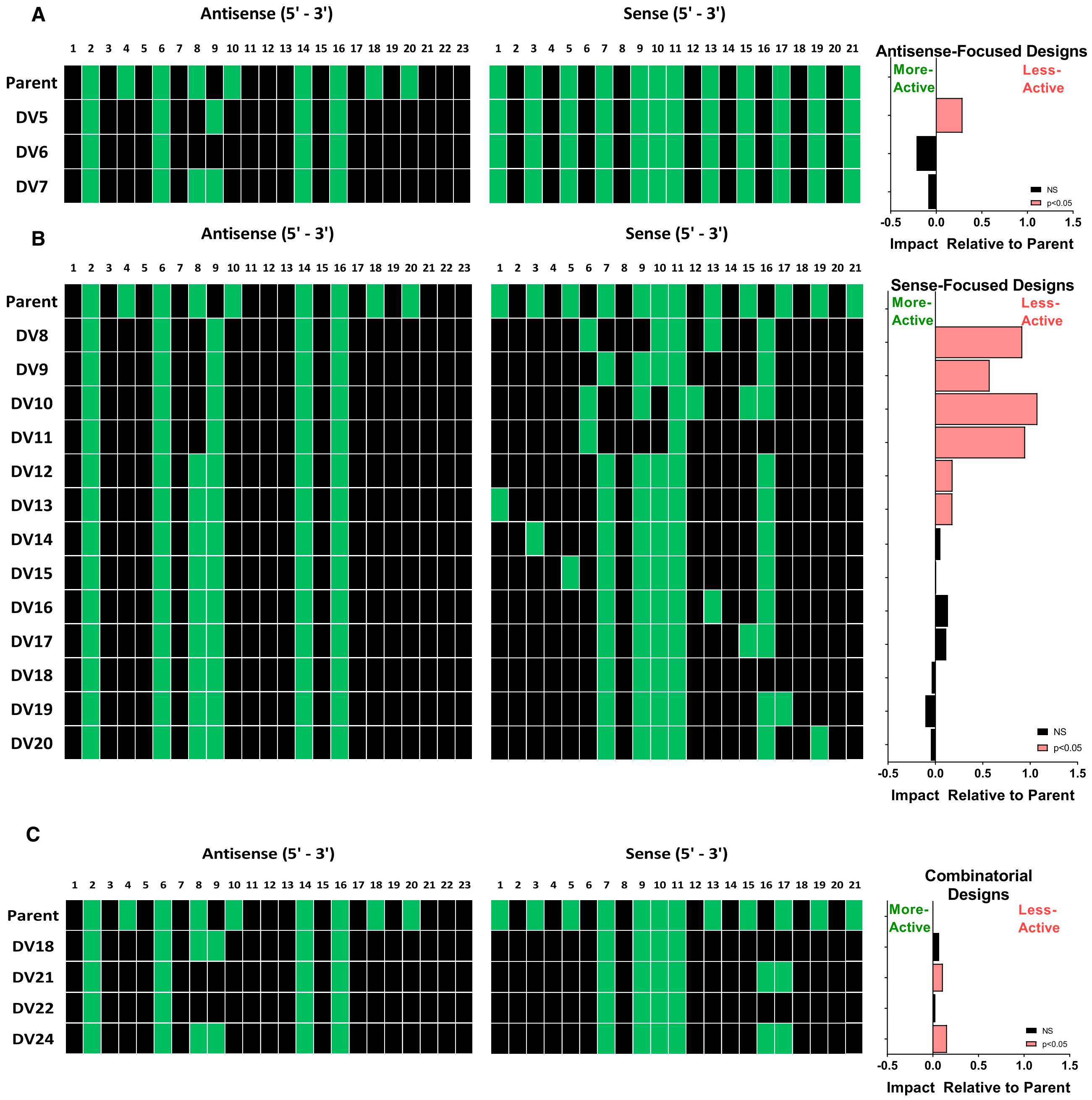

Figure 2. Sense and Antisense Strand Optimization

(A and B) Antisense (A) and sense (B) strand designs utilizing four siRNAs targeting the mouse transthyretin (*Ttr*) gene (Table S1) and the combination of best designs (C) evaluated across all 10 siRNAs (Table S1). Impact relative to parent is depicted as the model-adjusted mean difference in activity of design variant (DV) compared to parent, and it is presented in natural log. 2′-F and 2′-OMe modifications are depicted as green and black squares, respectively.

sequences, three each targeting mouse angiotensinogen (*Agt*) and three complement factor B (*Cfb*) (Figure 2C; Table S1). Across the larger panel of sequences, two of the four designs (DV 18 and DV 22), which shared a common sense design, maintained activity similar to the parent design.

Considering the extended exposure and enhanced nuclease activity *in vivo*, we rationalized that the benefit of improved metabolic stability due to increased 2′-OMe content would need to be evaluated *in vivo*. Hence, a critical part of the screening paradigm was to determine whether the *in vitro* optimization of the designs translated into

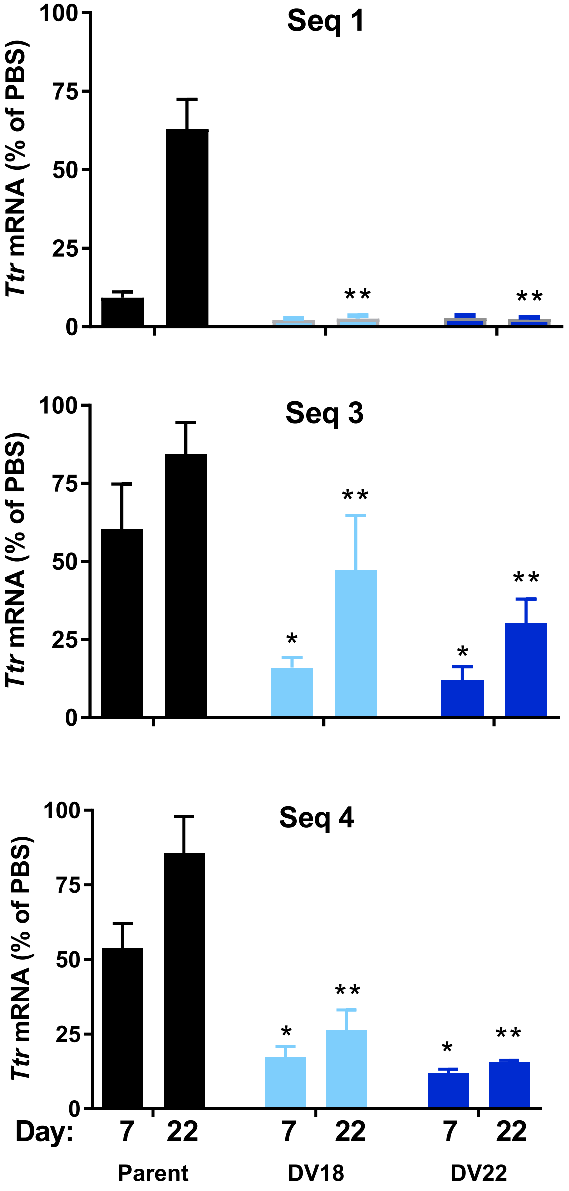

Figure 3. Application of Advanced Designs DV 18 and DV 22 to Multiple Sequences

In vivo activity and duration of optimized DVs compared to parent designs post single 3 mg/kg s.c. administration of conjugates in mice (n = 3 per group and time point). *Ttr* expression was assessed in liver at 7 and 22 days post-dose. Data are presented as average percent of PBS-treated animals. Data were analyzed by two-way ANOVA, followed by Dunnett's post-test for multiple comparisons. Results of post-test indicated as a single asterisk for significant compared to parent at day 7 and double asterisk for significant compared to parent at day 22. Error is represented as the SD.

improved *in vivo* performance and tissue exposure (as an indirect measure of stability). Silencing activity in mice was assessed for the best-performing template designs from the *in vitro* studies, DV 18 and DV 22, applied to three different siRNA sequences targeting mouse *Ttr* (Figure 3). Animals received 3 mg/kg GalNAc-siRNA conjugate or PBS, administered s.c., and *Ttr* expression was determined in liver by qPCR at days 7 and 22 post-dose. Across all three sequences tested, the new designs demonstrated increased efficacy and duration by two-way ANOVA compared to the parent ($p < 0.05$ for main effects, $p < 0.05$ for Dunnett's post-tests). The level of improvement was particularly pronounced at the later time point (day 22 post-dose), at which DV 18 reduced the levels of mRNA remaining in liver for the three sequences from 63.1%, 84.4%, and 85.6% observed for the parent to 6%, 47.3%, and 26.3%, respectively. Similarly, DV 22 reduced the *Ttr* mRNA levels to 2.5%, 30.4%, and 15.4%, respectively.

Translation across species was evaluated by applying one of the new designs (DV 22) to an siRNA targeting antithrombin (AT), which is fully cross-reactive with mouse, cynomolgus monkey, and human AT.[16] Relative to the parent, the optimized version yielded an increase in activity and duration in mice (Figure 4A) by two-way ANOVA ($p < 0.001$). DV 22 demonstrated improved activity at all time points except day zero and day 70 (Dunnett's post-test, $p < 0.05$ for each comparison).

Having confirmed improved efficacy and duration in mice, we evaluated these siRNAs in non-human primate to assess translation to a more clinically relevant species. Cynomolgus monkeys were treated with 1 mg/kg of the parent or corresponding analog with the DV 22 modification pattern. Consistent with the rodent data, the advanced design resulted in significantly enhanced efficacy and duration, demonstrating that the design improvements translate to higher species (Figure 4B). A two-way repeated-measures ANOVA indicated a significant effect of design (DV 22 versus parent, $p = 0.0004$), with the difference apparent beginning at day 8 post-dose and continuing through study end (Sidak's multiple comparison test, $p < 0.05$). Comparison of the recovery portion of the curves (days 22 through 71) by linear regression revealed a statistically significant difference in slope (0.92% versus 0.57% of control per day, for parent and DV 22, respectively) as well as an ~2-fold enhanced target silencing by area under the curve (AUC values of 4,168 and 9,064 for the parent and DV 22, respectively).

Next, we aimed to confirm our initial hypothesis and demonstrate that the improvements to *in vivo* performance observed with the new designs were predominantly due to enhanced metabolic stability rather than enhanced interactions with the RNAi machinery. Therefore, a set of GalNAc-siRNA conjugates with increasing 2′-OMe content (parent, DV 18, and fully 2′-OMe) was compared in mice measuring target silencing and siRNA levels in total liver as well as Argonaute 2 (Ago2)-associated siRNA over a period of 35 days following a single s.c. dose. To achieve similar levels of target silencing at nadir, the parent conjugate was dosed at 2.5 mg/kg, while the DV 18 and the inactive full 2′-OMe control were dosed at 0.75 mg/kg. Cohorts of animals (n = 3/group/time point) were sacrificed at 4 hr and days 3, 7, 14, 21, and 35, and *Ttr* mRNA knockdown, total siRNA liver levels, and the amount of Ago2-loaded

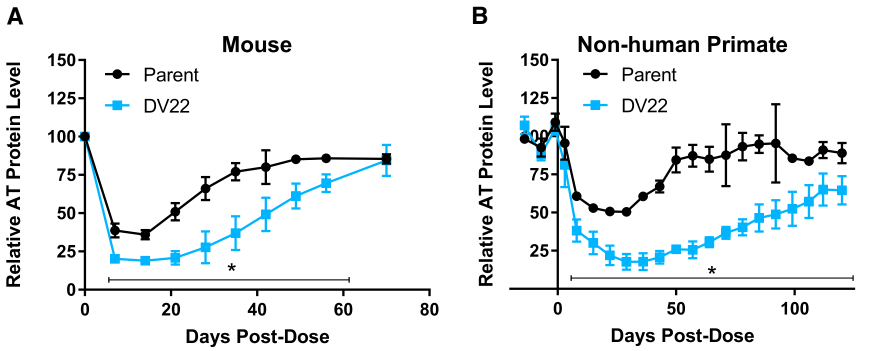

Figure 4. Application of New Design DV 22 to siRNA-Targeting AT

(A) Mice (n = 3 per group) were treated with a single dose (1 mg/kg) of parent or the DV 22-based derivative. Serum AT levels were assessed by ELISA. (B) Cynomolgus monkeys (n = 3 per group) were treated with a single dose (1 mg/kg) of parent or its DV 22-based derivative, and plasma AT levels were assessed by AT activity assay. Data were analyzed by two-way ANOVA, followed by Dunnett's (A) or Sidak's (B) post-test. Time points at which a significant difference was observed between parent and DV 22 are noted with an asterisk. Error is represented as the SD.

sense and antisense strands were measured at each time point (Figure 5).

The fully 2′-OMe-modified control showed the highest exposure in the liver followed by the DV 18-based conjugate, which showed a 1.7-fold increase in liver exposure over its parent despite an ∼3-fold lower dose (Figure 5A). As expected, no target silencing was observed in animals that received the fully 2′-OMe-modified conjugate, while robust *Ttr* mRNA knockdown was observed with parent and DV 18 conjugates (Figures 5B–5D). Animals dosed with the parent conjugate achieved up to 92% *Ttr* knockdown on day 3 followed by a rapid recovery (∼19% knockdown, day 35). In contrast, a 3-fold lower dose of the DV 18 conjugate sustained ∼85% knockdown from days 3 to 21, followed by a slower recovery (70% knockdown, day 35). Target silencing profiles were found to strongly correlate with the levels of Ago2-loaded antisense strands, confirming previous observations on siRNA pharmacokinetic/pharmacodynamic (PK/PD).[17] Ago2 loading for the antisense strand of the parent conjugate peaked at day 3 followed by a relatively steep decline, reaching background levels at the end of the study. In contrast, Ago2 loading in the DV 18 group plateaued at day 7 with sustained levels until day 14 before decreasing. Sense strand loading throughout the time course was barely above background detection limits except for a small peak on day 3 in the parent group. As expected and despite having the highest liver exposure among the three conjugates tested, fully 2′-OMe-modified conjugates exhibited very low Ago2 loading for both sense and antisense strands, in agreement with the lack of *Ttr* silencing.

The relationship between Ago2 antisense loading and *Ttr* silencing is depicted in Figure 5E. The results indicate that the absolute amount of loaded antisense strand in Ago2 needed to achieve a certain level of silencing was similar between the parent and DV 18 designs, with half-maximal effective concentration (EC_{50}) calculated to be 0.066 ng/g and 0.046 ng/g and EC80 of 0.24 ng/g and 0.20 ng/g for parent and DV 18, respectively. The data were fitted to semi-log curves and compared by extra sum of squares F-test, revealing the lines to be indistinguishable ($p = 0.31$). Taken together, these data further support the hypothesis that the *in vivo* benefit seen with advanced design conjugates predominantly stems from enhanced metabolic stability, leading to improved liver exposure, rather than from differences in inherent RNAi activity.

DISCUSSION

Since the discovery of siRNA in 2001, researchers have made great progress toward harnessing RNAi as a therapeutic modality, with several programs in late-stage clinical development.[4,28] For a conjugate approach utilizing a targeting ligand directly connected to the siRNA, one of the key challenges has been to adequately protect the siRNA against nucleolytic degradation while maintaining its ability to interact with RISC for efficient target cleavage. Our studies have shown that relatively small changes in design can have a large impact on metabolic stability, thereby affecting the *in vivo* performance of the siRNA conjugates.[17] Based on the hypothesis that additional enhancements in metabolic stability could further improve *in vivo* performance, we explored whether the design of fully 2′-F- and 2′-OMe-modified conjugates could be further optimized. The aim here was to carefully evaluate positioning and balance between the two ribosugar modifications, which had not yet been refined for optimal *in vivo* performance.

A screening paradigm was developed based on the statistical analysis of a large *in vitro* dataset to guide initial DVs, which utilizes parallel evaluation of new designs across multiple sequences. Using this approach, we were able to identify and confirm critical positions in both strands, which require the smaller 2′-F rather than the sterically more demanding 2′-OMe modification to maintain potency. Our model did not include interactions between variables since the statistical evaluation of such interactions would require a substantially larger dataset. However, some of the discrepancies observed between modeling and experimental results suggest that the positional effect of 2′ modifications on activity cannot be considered in isolation but may well be sensitive to the context, i.e., the neighboring modifications and even the overall modification pattern of the siRNA. Initial screens, for instance, relied on the separate evaluation of antisense and sense strands, but the results indicated that the performance ultimately depended on the specific combination of sense and antisense strand designs. This suggests a degree of chemistry interdependence across the complementary strands of the siRNA duplex for optimal silencing activity.

Combinations of the most potent sense and antisense strand designs were evaluated across a broader set of sequences. Two designs, DV 18 and DV 22, were identified that met the criteria of low 2′-F content while maintaining *in vitro* activity across a large panel of sequences, confirming the generalizability of the advanced designs. Importantly,

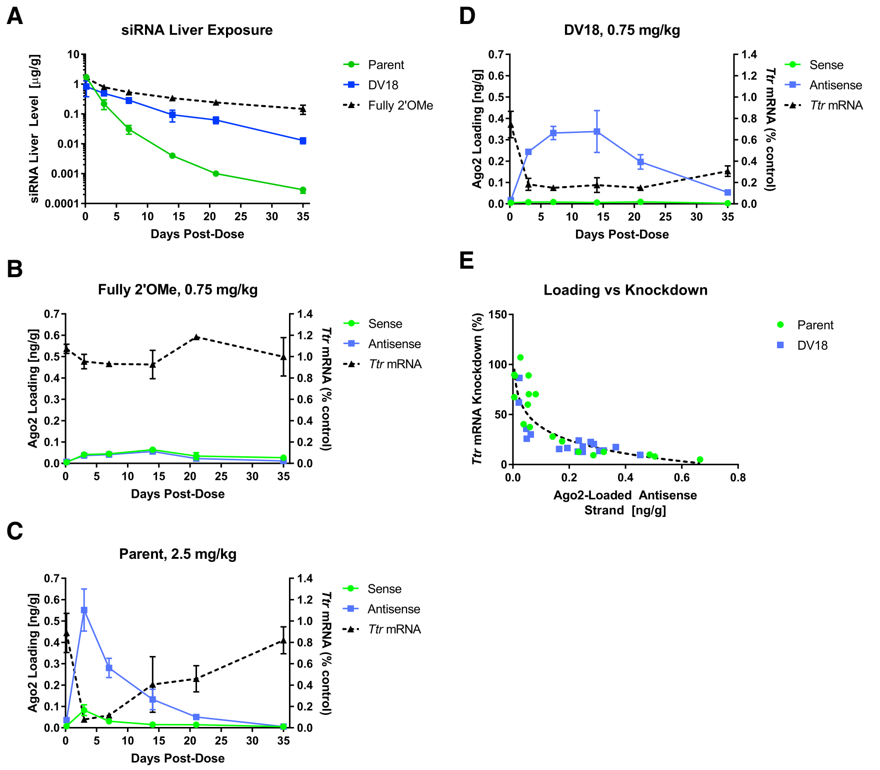

Figure 5. *In Vivo* Evaluation of Parent, DV 18, and Fully 2′-OMe Conjugates Targeting *Ttr*

(A) Time course analysis of antisense strand liver levels (µg/g, microgram antisense strand per gram of liver) from mouse cohorts (n = 3/group/time) dosed with Parent (green line, 2.5 mg/kg), DV 18 (blue line, 0.75 mg/kg), or fully 2′-OMe (black dashed line, 0.75 mg/kg) conjugates targeting *Ttr*. (B–D) Time course analyses of *Ttr* mRNA knockdown (dashed black line) and sense (green line) and antisense (blue line) Ago2 loading (ng/g, nanogram sense or antisense strand per gram liver) for parent (B), DV 18 (C), and fully 2′-OMe (D) conjugates. *Ttr* mRNA levels were normalized to *Gapdh* mRNA for each animal and then to PBS control animals. (E) Comparison plot of Ago2-loaded antisense with *Ttr* mRNA knockdown for parent (green circles) and DV 18 (blue squares) conjugates. Semi-log lines of best fit are overlaid. Error is represented as the SD.

both designs showed substantially improved *in vivo* efficacy and duration, suggesting that the refinement of the siRNA chemistry translated into enhanced metabolic stability.[29]

With species-dependent differences in nuclease activities across the various extracellular and intracellular compartments, an understanding of the translation across species is fundamental to supporting the advancement of novel designs into development. Hence, we investigated the performance of a cross-reactive siRNA targeting AT in mouse and non-human primate (NHP). The results indicate that the improvement is similar in both species, with increased efficacy as well as duration.

Detailed analysis of *in vitro* and *in vivo* potency, total liver levels, and Ago2-loaded antisense strand demonstrate that the benefits of advanced designs in terms of conjugate efficacy and duration predominantly appear to be due to an increase in metabolic stability of GalNAc-siRNA conjugates, which provides a larger pool of active siRNA that can be incorporated into the cellular RNAi machinery over time. This confirms our hypothesis that the *in vivo* performance of ESC conjugates could be further enhanced by optimizing the 2′-F/2′-OMe modification pattern. Specifically, this was achieved by a >50% reduction in 2′-F content (and a concomitant increase in 2′-OMe content) across both strands of the siRNA without compromising the intrinsic activity.

Taken together, we developed a screening paradigm for the optimization of siRNA designs, which is based on a positional model for chemical modifications and relies on parallel screening across a larger set of sequences and *in vivo* evaluation as part of the process. This method was successfully applied to further improve the metabolic stability of siRNA conjugates. The new designs feature a substantial reduction in 2′-F content while maintaining *in vitro* activity across a large panel of sequences and showing excellent *in vivo* performance. The screening paradigm reported here is well suited to systematically evaluate novel nucleic acid chemistries, which may help to identify opportunities for further improvements in siRNA design, thereby expanding the therapeutic potential of RNAi therapeutics.

MATERIALS AND METHODS

siRNA Synthesis

All oligonucleotides were prepared on a 1-µmol scale using a MerMade 192 high-throughput synthesizer and commercially available 5′-O-DMT-3′-O-2-cyanoethyl phosphoramidite monomers, following standard protocols for solid-phase synthesis and deprotection.[30,31] The GalNAc ligand was introduced at the 3′ end of the sense strand of the siRNA using a functionalized solid support, as previously described.[14] PS linkages were introduced between antisense nucleotides 1 and 2, 2 and 3, 21 and 22, and 22 and 23 from the 5′ end, as well as sense nucleotides 1 and 2 and 2 and 3 from the 5′ end. PS linkages were prepared by oxidation of phosphite utilizing 0.1 M 3-((*N*,*N*-dimethyl-aminomethylidene)amino)-3H-1, 2, 4-dithiazole-5-thione (DDTT) in pyridine. After cleavage, deprotection, and precipitation of the products, each crude solution was desalted via size exclusion using water to elute the final oligonucleotide products. The identities and purities of all oligonucleotides were confirmed using electrospray ionization mass spectrometry (ESI-MS) and ion exchange-high-performance liquid chromatography (IEX-HPLC), respectively, and equimolar amounts of the complementary strands were annealed to provide the desired siRNA duplex. All duplexes met a purity cutoff of at least 75%.

Statistical Analysis

Target-silencing data from a series of design efforts were compiled for analysis in R. Data were expressed as fold change (relative to 1), and mRNA target sites were assigned a unique identifier to control for multiple designs at a given site in a transcript. Duplex chemistries were then expressed independently of nucleobase composition (i.e., 2′-Fluoro instead of 2′-deoxy-2′-fluorouridine) to better enable comparison across sequences. To enrich the analysis for target sites on which multiple designs were evaluated, we restricted the analysis to target sites for which at least ten designs were evaluated. The dataset then consisted of 15 target sites across five targets. Note that analyses were agnostic to terminal PS content. The final analysis consisted of data derived from transfection studies at 0.1 nM, approximately 2,000 data points. A multiple linear regression was performed using the statistical programming language R (https://cran.r-project.org/), with variables of target, target site identifier, species of cell culture line, and chemistry at each position in the duplex. 2′-OMe was considered the reference value for chemistry, for the purpose of comparison.

For analyses on directed design efforts, target-silencing data were collected from cells treated at 10 and 0.1 nM, as described. Each target site was assigned a unique identifier and similarly each DV was assigned an identifier. Data were analyzed by multiple linear regression, using log-transformed target-silencing data. Variables evaluated included concentration of siRNA, target site identifier, and replicate number (to control for systematic effects in studies). The comparator for the analysis (such as parent variant) was assigned as the reference value for the DV.

The coefficients of the above models represent the model-adjusted mean difference, in natural log of the comparator (such as 2′-F or a novel design) from the reference design (such as 2′-OMe). Coefficients with an associated p value of less than 0.05 were considered significant.

Statistical analyses of mouse and non-human primate data were performed with GraphPad Prism 7. Mouse-silencing activity data were evaluated by two-way ANOVA with Dunnett's post-test for multiple comparisons, with main effects of time and design. Curve fits for the association of Ago2 loading with target silencing were accomplished by semilog linear regressions (with log-transformed Ago2 loading) and lines compared by extra sum of squares F-test. For non-human primate, the repeated-measures ANOVA (with main effects of time and design) was performed with multiple comparisons for assessing differences at each time point, using Sidak's test for correction. Comparison of the return to baseline was evaluated by linear regression on data from days 29 through 71 post-dose with comparison of lines, which were approximately linear. AUC was evaluated using a baseline value of 100, ignoring peaks less than 10% of the distance from minimum to maximum Y and including peaks that went below baseline. For all tests, a significance threshold of $p < 0.05$ was observed.

Cell Culture

Cryopreserved primary mouse hepatocytes (Thermo Fisher Scientific, various lots) were cultured in Williams E media supplemented with 10% fetal bovine serum (Thermo Fisher Scientific, 16140071) on collagen-coated 384-well plates (356666). Cells were transfected with siRNA at either 10 or 0.1 nM final concentration, using RNAiMax according to the manufacturer's protocol (Thermo Fisher Scientific, 13778100). All transfections were carried out in quadruplicate. Prior to RNA isolation, cells were incubated at 37°C in 5% CO_2 for 20–24 hr.

The mRNA was isolated using an automated 384-well protocol. Briefly, media were removed from cells and replaced with lysis buffer, and cells were lysed in plate using an electromagnetic shaker. Following lysis, mRNA capture and washing were performed using a BioTek plate washer, following a custom magnetic bead purification protocol using Dynabeads mRNA DIRECT Purification Kit (Thermo Fisher Scientific, 61012). Following washing, wash buffer was removed and replaced with 10 μL RNase-free water.

The cDNA synthesis was performed using a high-capacity cDNA reverse transcription kit (4368814) using 10 μL isolated mRNA as a template. Reactions were performed according to the protocol and incubated at 37°C for 2 hr. Following incubation, plates were heated to inactivate the enzyme and 2 μL of the product was used as a template for qPCR.

The qPCR reactions were performed in multiplex using a gene-specific TaqMan assay for each target (Thermo Fisher Scientific, Mm00443267_m1 [*Ttr*], Mm00433918_g1 [*Cfb*], and Mm00599662_m1 [*Agt*]) and mouse *Gapdh* as an endogenous control (4352339E). Real-Time PCR was performed in a Roche LightCycler 480 using LightCycler 480 Probes Master Mix (Roche, 04707494001). Data were analyzed using the ΔΔCt method normalizing to cells transfected with a non-targeting negative control.

In Vivo Experiments

In vivo experiments were conducted by certified laboratory personnel using protocols consistent with local, state, and federal regulations. Experimental protocols were approved by the Institutional Animal Care and Use Committee and the Association for Assessment and Accreditation of Laboratory Animal Care International (accreditation 001345). The 8-week-old C57BL/6 female mice were obtained from Charles River Laboratories. Mice were maintained on *ad libitum* food and water. For evaluation of *Ttr*, mice (n = 3 per group and time point) received PBS or siRNA by s.c. injection in a volume of 10 ml/kg, at a dose of 3 mg/kg. At days 7 and 22 post-dose, three mice per group were euthanized with carbon dioxide. Liver was harvested and snap-frozen in liquid nitrogen. The liver tissues were processed in a SPEX GenoGrinder (250 strokes per second, 1× speed, 120 s) with one stainless steel ball per collection cup. A small amount of each tissue was transferred to Matrix Storage Tubes (Thermo Fisher Scientific, 4252) on dry ice for lysis. RNA extraction was performed with a PureLink Pro 96 total RNA Purification Kit (Life Technologies). Reverse transcription and qPCR were conducted as described for *in vitro* studies. Mm00443267_m1 was used to quantify mouse *Ttr*, with mouse *Gapdh* (4352339E) as the loading control.

For AT silencing in mice (n = 3 per group), blood was first obtained from the animals by retroorbital bleed, and mice subsequently received siRNA (prepared as described for *Ttr*). Animals were then bled every 7 days through day 70 post-dose. At the times indicated, blood was processed to obtain plasma. AT levels were determined by ELISA (Abcam, Ab108800). Data were normalized to pre-dose values.

Non-human primate studies were performed by Covance, using cynomolgus monkeys. Male cynomolgus monkeys (2–5 kg in weight) were housed with *ad libitum* access to food and water, as well as appropriate enrichment. At the indicated time points, blood was obtained and processed to serum. AT levels were assessed by activity assay (Ariana, A221105). Data were normalized to pre-dose values.

siRNA Pharmacokinetics

C57BL/6 male mice, aged 6–8 weeks, were acquired from Charles River Laboratories and housed with *ad libitum* access to food and water. Mice were dosed s.c. with a volume of 10 μL per gram of body weight (n = 3 per time point) at a dose of either 0.75 mg/kg (*Ttr*-targeting parent variant and 2′-OMe conjugates) or at 2.5 mg/kg (DV 18). The control group received PBS. Mice were not fasted. Livers (n = 3/time point/group) were taken at specified time points. *Ttr* mRNA levels were quantified using methods described earlier.[14]

Quantification of Whole Liver and Ago2-Associated siRNA Levels

Mice were sacrificed on day 7 post-dose, and livers were snap-frozen in liquid nitrogen and ground into powder for further analysis. Total siRNA liver levels were measured based on previously published methods. Ago2-bound siRNA from mouse liver was quantified by stem-loop (SL)-qPCR as described. For SL-qPCR, the following probes and primers were used: sense stem loop primer GTCGTATC CAGTGCAGGGTCCGAGGTATTCGCACTGGATACGACTATA GAGCAA, sense forward primer gccgcgcAACAGTGTTCTTG, sense probe CTGGATACGACTATAGAGC, antisense stem loop primer GTCGTATCCAGTGCAGGGTCCGAGGTATTCGCACTGGATA CGACAAAACAGTGT, antisense forward primer gccgcgcTTATAG AGCAAG, antisense probe CTGGATACGACAAAACAGT, and universal reverse primer GTGCAGGGTCCGAGGT.

SUPPLEMENTAL INFORMATION

Supplemental Information includes one figure and one table and can be found with this article online at https://doi.org/10.1016/j.ymthe.2017.12.021.

AUTHOR CONTRIBUTIONS

D.J.F., C.R.B., K.Q., and A.S. designed and performed experiments. S.S., C.T., M.K.S., and S.K. generated reagents and carried out siRNA synthesis. S.K., K.G.R., M.M., and V.J. provided experimental and strategic guidance. M.A.M., S.M., D.J.F., and C.R.B. designed experiments and wrote the manuscript.

CONFLICTS OF INTEREST

All authors are, or were during the time this work was conducted, employees of Alnylam Pharmaceuticals.

ACKNOWLEDGMENTS

We thank John Maraganore, Rachel Meyers, Klaus Charisse, Akin Akinc, Greg Hinkle, and Kevin Fitzgerald for helpful discussions and Sean Monahan for help with coding.

REFERENCES

1. Elbashir, S.M., Harborth, J., Lendeckel, W., Yalcin, A., Weber, K., and Tuschl, T. (2001). Duplexes of 21-nucleotide RNAs mediate RNA interference in cultured mammalian cells. Nature *411*, 494–498.
2. Fire, A., Xu, S., Montgomery, M.K., Kostas, S.A., Driver, S.E., and Mello, C.C. (1998). Potent and specific genetic interference by double-stranded RNA in Caenorhabditis elegans. Nature *391*, 806–811.
3. Hannon, G.J. (2002). RNA interference. Nature *418*, 244–251.
4. Bobbin, M.L., and Rossi, J.J. (2016). RNA Interference (RNAi)-Based Therapeutics: Delivering on the Promise? Annu. Rev. Pharmacol. Toxicol. *56*, 103–122.
5. Coelho, T., Adams, D., Silva, A., Lozeron, P., Hawkins, P.N., Mant, T., Perez, J., Chiesa, J., Warrington, S., Tranter, E., et al. (2013). Safety and efficacy of RNAi therapy for transthyretin amyloidosis. N. Engl. J. Med. *369*, 819–829.
6. Fitzgerald, K., Frank-Kamenetsky, M., Shulga-Morskaya, S., Liebow, A., Bettencourt, B.R., Sutherland, J.E., Hutabarat, R.M., Clausen, V.A., Karsten, V., Cehelsky, J., et al. (2014). Effect of an RNA interference drug on the synthesis of proprotein convertase subtilisin/kexin type 9 (PCSK9) and the concentration of serum LDL cholesterol in healthy volunteers: a randomised, single-blind, placebo-controlled, phase 1 trial. Lancet *383*, 60–68.
7. Frank-Kamenetsky, M., Grefhorst, A., Anderson, N.N., Racie, T.S., Bramlage, B., Akinc, A., Butler, D., Charisse, K., Dorkin, R., Fan, Y., et al. (2008). Therapeutic RNAi targeting PCSK9 acutely lowers plasma cholesterol in rodents and LDL cholesterol in nonhuman primates. Proc. Natl. Acad. Sci. USA *105*, 11915–11920.
8. Matsuda, S., Keiser, K., Nair, J.K., Charisse, K., Manoharan, R.M., Kretschmer, P., Peng, C.G., V Kel'in, A., Kandasamy, P., Willoughby, J.L., et al. (2015). siRNA conjugates carrying sequentially assembled trivalent N-acetylgalactosamine linked through nucleosides elicit robust gene silencing in vivo in hepatocytes. ACS Chem. Biol. *10*, 1181–1187.
9. Grewal, P.K., Uchiyama, S., Ditto, D., Varki, N., Le, D.T., Nizet, V., and Marth, J.D. (2008). The Ashwell receptor mitigates the lethal coagulopathy of sepsis. Nat. Med. *14*, 648–655.
10. Khorev, O., Stokmaier, D., Schwardt, O., Cutting, B., and Ernst, B. (2008). Trivalent, Gal/GalNAc-containing ligands designed for the asialoglycoprotein receptor. Bioorg. Med. Chem. *16*, 5216–5231.
11. Pricer, W.E., Jr., Hudgin, R.L., Ashwell, G., Stockert, R.J., and Morell, A.G. (1974). A membrane receptor protein for asialoglycoproteins. Methods Enzymol. *34*, 688–691.
12. Rensen, P.C., van Leeuwen, S.H., Sliedregt, L.A., van Berkel, T.J., and Biessen, E.A. (2004). Design and synthesis of novel N-acetylgalactosamine-terminated glycolipids for targeting of lipoproteins to the hepatic asialoglycoprotein receptor. J. Med. Chem. *47*, 5798–5808.
13. Valentijn, A.R.P.M., van der Marel, G.A., Sliedregt, L.A.J.M., van Berkel, T.J.C., Biessen, E.A.L., and van Boom, J.H. (1997). Solid-phase synthesis of lysine-based cluster galactosides with high affinity for the asialoglycoprotein Receptor. Tetrahedron *53*, 759–770.
14. Nair, J.K., Willoughby, J.L., Chan, A., Charisse, K., Alam, M.R., Wang, Q., Hoekstra, M., Kandasamy, P., Kel'in, A.V., Milstein, S., et al. (2014). Multivalent N-acetylgalactosamine-conjugated siRNA localizes in hepatocytes and elicits robust RNAi-mediated gene silencing. J. Am. Chem. Soc. *136*, 16958–16961.
15. Chan, A., Liebow, A., Yasuda, M., Gan, L., Racie, T., Maier, M., Kuchimanchi, S., Foster, D., Milstein, S., Charisse, K., et al. (2015). Preclinical Development of a Subcutaneous ALAS1 RNAi Therapeutic for Treatment of Hepatic Porphyrias Using Circulating RNA Quantification. Mol. Ther. Nucleic Acids *4*, e263.

16. Sehgal, A., Barros, S., Ivanciu, L., Cooley, B., Qin, J., Racie, T., Hettinger, J., Carioto, M., Jiang, Y., Brodsky, J., et al. (2015). An RNAi therapeutic targeting antithrombin to rebalance the coagulation system and promote hemostasis in hemophilia. Nat. Med. *21*, 492–497.

17. Nair, J., Attarwala, H., Sehgal, A., Wang, Q., Aluri, K., Zhang, X., Gao, M., Liu, J., Indrakanti, R., Schofield, S., et al. (2017). Impact of enhanced metabolic stability on pharmacokinetics and pharmacodynamics of GalNAc-siRNA conjugates. Nucleic Acids Res. *45*, 10969–10977.

18. Cummins, L.L., Owens, S.R., Risen, L.M., Lesnik, E.A., Freier, S.M., McGee, D., Guinosso, C.J., and Cook, P.D. (1995). Characterization of fully 2′-modified oligoribonucleotide hetero- and homoduplex hybridization and nuclease sensitivity. Nucleic Acids Res. *23*, 2019–2024.

19. Takahashi, M., Minakawa, N., and Matsuda, A. (2009). Synthesis and characterization of 2′-modified-4′-thioRNA: a comprehensive comparison of nuclease stability. Nucleic Acids Res. *37*, 1353–1362.

20. Chiu, Y.L., and Rana, T.M. (2003). siRNA function in RNAi: a chemical modification analysis. RNA *9*, 1034–1048.

21. Manoharan, M., Akinc, A., Pandey, R.K., Qin, J., Hadwiger, P., John, M., Mills, K., Charisse, K., Maier, M.A., Nechev, L., et al. (2011). Unique gene-silencing and structural properties of 2′-fluoro-modified siRNAs. Angew. Chem. Int. Ed. Engl. *50*, 2284–2288.

22. Prakash, T.P., Allerson, C.R., Dande, P., Vickers, T.A., Sioufi, N., Jarres, R., Baker, B.F., Swayze, E.E., Griffey, R.H., and Bhat, B. (2005). Positional effect of chemical modifications on short interference RNA activity in mammalian cells. J. Med. Chem. *48*, 4247–4253.

23. Janas, M.M., Jiang, Y., Schlegel, M.K., Waldron, S., Kuchimanchi, S., and Barros, S.A. (2017). Impact of Oligonucleotide Structure, Chemistry, and Delivery Method on In Vitro Cytotoxicity. Nucleic Acid Ther. *27*, 11–22.

24. Janas, M.M., Jiang, Y., Duncan, R.G., Hayes, A.N., Liu, J., Kasperkovitz, P.V., Placke, M.E., and Barros, S.A. (2016). Exposure to siRNA-GalNAc Conjugates in Systems of the Standard Test Battery for Genotoxicity. Nucleic Acid Ther. *26*, 363–371.

25. Pasi, K.J., Rangarajan, S., Georgiev, P., Mant, T., Creagh, M.D., Lissitchkov, T., Bevan, D., Austin, S., Hay, C.R., Hegemann, I., et al. (2017). Targeting of Antithrombin in Hemophilia A or B with RNAi Therapy. N. Engl. J. Med. *377*, 819–828.

26. Shen, W., Liang, X.H., Sun, H., and Crooke, S.T. (2015). 2′-Fluoro-modified phosphorothioate oligonucleotide can cause rapid degradation of P54nrb and PSF. Nucleic Acids Res. *43*, 4569–4578.

27. Fitzgerald, K., White, S., Borodovsky, A., Bettencourt, B.R., Strahs, A., Clausen, V., Wijngaard, P., Horton, J.D., Taubel, J., Brooks, A., et al. (2017). A Highly Durable RNAi Therapeutic Inhibitor of PCSK9. N. Engl. J. Med. *376*, 41–51.

28. Wittrup, A., and Lieberman, J. (2015). Knocking down disease: a progress report on siRNA therapeutics. Nat. Rev. Genet. *16*, 543–552.

29. Bramsen, J.B., Laursen, M.B., Nielsen, A.F., Hansen, T.B., Bus, C., Langkjaer, N., Babu, B.R., Højland, T., Abramov, M., Van Aerschot, A., et al. (2009). A large-scale chemical modification screen identifies design rules to generate siRNAs with high activity, high stability and low toxicity. Nucleic Acids Res. *37*, 2867–2881.

30. Beaucage, S.L. (2008). Solid-phase synthesis of siRNA oligonucleotides. Curr. Opin. Drug Discov. Devel. *11*, 203–216.

31. Mueller, S., Wolf, J., and Ivanov, S.A. (2004). Current Strategies for the Synthesis of RNA. Curr. Org. Synth. *1*, 293–307.

YMTHE, Volume *26*

Supplemental Information

Advanced siRNA Designs Further Improve *In Vivo* Performance of GalNAc-siRNA Conjugates

Donald J. Foster, Christopher R. Brown, Sarfraz Shaikh, Casey Trapp, Mark K. Schlegel, Kun Qian, Alfica Sehgal, Kallanthottathil G. Rajeev, Vasant Jadhav, Muthiah Manoharan, Satya Kuchimanchi, Martin A. Maier, and Stuart Milstein

Supplementary Information

Advanced siRNA designs further improve *in vivo* performance of GalNAc-siRNA conjugates

Donald J Foster, Christopher R Brown, Sarfraz Shaikh, Casey Trapp, Mark K. Schlegel, Kun Qian, Alfica Sehgal, Kallanthottathil G. Rajeev, Vasant Jadhav, Muthiah Manoharan, Satya Kuchimanchi, Martin A. Maier, and Stuart Milstein[*]

Content

Supplementary Table 1. Sequence information of siRNAs used in this work

Supplementary Figure 1. Alternate analysis of optimization of sense and antisense strand designs

Supplementary Table 1

Target	Sequence	Strand	Sequence (5’ – 3’)
Mouse *Ttr* (NM_013697.5)	1	S	AACAGUGUUCUUGCUCUAUAA
		AS	UUAUAGAGCAAGAACACUGUUUU
Mouse *Ttr* (NM_013697.5)	2	S	AAACAGUGUUCUUGCUCUAUA
		AS	UAUAGAGCAAGAACACUGUUUUG
Mouse *Ttr* (NM_013697.5)	3	S	CUUGCUCUAUAAACCGUGUUA
		AS	UAACACGGUUUAUAGAGCAAGAA
Mouse *Ttr* (NM_013697.5)	4	S	UCCUCUGAUGGUCAAAGUCCU
		AS	AGGACUUUGACCAUCAGAGGACA
Mouse *Cfb* (NM_008198.2)	5	S	GAUUGAGAAGGUGGCGAGUUA
		AS	UAACUCGCCACCUUCUCAAUCAA
Mouse *Cfb* (NM_008198.2)	6	S	CACAGAGAAGCUCAACCAAAU
		AS	AUUUGGUUGAGCUUCUCUGUGAC
Mouse *Cfb* (NM_008198.2)	7	S	UUGUGAGAGAGAUGCUACAAA
		AS	UUUGUAGCAUCUCUCUCACAACU
Mouse *Agt* (NM_007428.3)	8	S	GCGCUGAAGGAUACACAGAAA
		AS	UUUCUGUGUAUCCUUCAGCGCCA
Mouse *Agt* (NM_007428.3)	9	S	ACUGCGCUGACCGAGAAUAAA
		AS	UUUAUUCUCGGUCAGCGCAGUCU
Mouse *Agt* (NM_007428.3)	10	S	CCGAGUGGGAGAGGUUCUCAA
		AS	UUGAGAACCUCUCCCACUCGGGG

Supplementary Figure 1. Alternate analysis of optimization of sense and antisense strand designs. Impact relative to DV5 (A, B) or DV12 (C) is defined as the model-adjusted mean difference in activity of design variant (DV) compared to reference, and is presented in natural log.

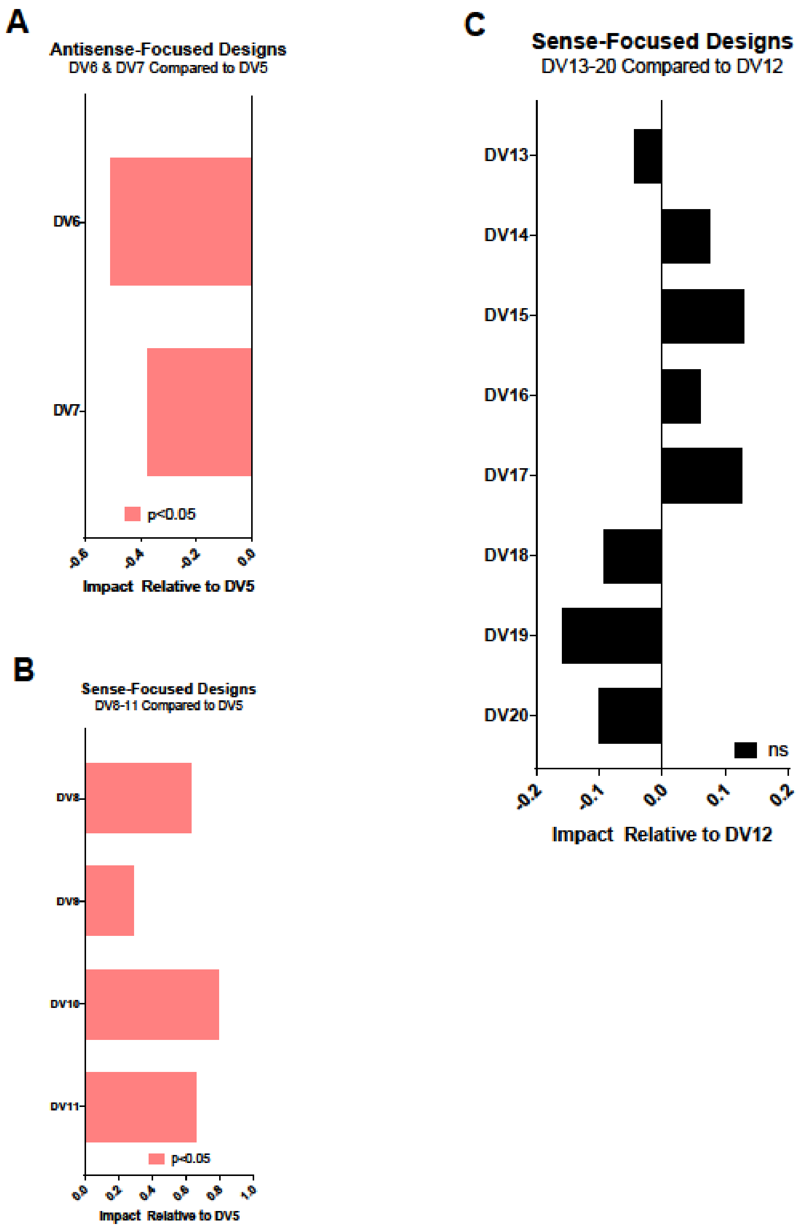

Supplement: Document S2. Article plus Supplemental Information [file mmc2.pdf]
